# Easing pandemic-related restrictions, easing psychosocial stress factors in families with infants and toddlers? Cross-sectional results of the three wave CoronabaBY study from Germany

**DOI:** 10.1186/s13034-023-00618-7

**Published:** 2023-06-23

**Authors:** Anna Friedmann, Catherine Buechel, Clara Seifert, Stefan Eber, Volker Mall, Ina Nehring

**Affiliations:** 1 Chair of Social Pediatrics, TUM School of Medicine, Technical University of Munich, Heiglhofstraße 65, 81377 Munich, Germany; 2 Chair of Human Movement Science, Department of Sport and Health Sciences, Technical University of Munich, Munich, Germany; 3 Professional Association of Pediatricians in Bavaria (BVKJ) and PaedNetz Bayern, Munich, Germany

**Keywords:** Infant mental health, COVID-19 pandemic, Depression, Anxiety, Parenting stress

## Abstract

**Background:**

Families with young children are particularly vulnerable for the stressors induced by the COVID-19 pandemic. However, studies on their psychosocial situation during the course of the crisis are still sparse.

**Methods:**

In a comparison of three survey waves (wave I and III = high COVID-19 incidences), we cross-sectionally investigated the proportion of families (N_total_ = 2940) with children aged 0–3 years experiencing pandemic burden, parenting stress, and parental and child mental health problems in relation to COVID-19 incidences and restrictions in Southern Germany via validated questionnaires. Potential influencing factors were also explored.

**Results:**

The number of parents with a high pandemic burden decreased over the course of the pandemic with a peak of 65.3% in wave I (significant changes except wave II versus III). Participants with high parenting stress significantly increased from 38.2% in wave I to 51.2% in wave III. The number of parents with symptoms of depression and anxiety remained constantly high with a maximum of 28.4% being affected. Infants with crying/sleeping problems increased significantly from 26.4% in wave I to 35.5% in wave III. Toddlers’ emotional and behavioral problems showed a peak of 23.9% in wave III (no significant changes). Increased family conflicts were the strongest predictor for parenting stress (ß = 0.355), maternal (ß = 0.305), infants’ (ß = 0.149) and toddlers’ (ß = 0.216) mental health problems during the pandemic.

**Conclusions:**

Psychosocial stress factors in families with infants and toddlers remained highly pronounced and even partly increased irrespective of pandemic events. The findings suggest a staggered negative impact of pandemic-related factors on young children’s mental health. Promoting infants’ mental health as well as strengthening parental resources by reducing parenting stress should be a top healthcare priority in the aftermath of COVID-19.

*Trial registration* The study was pre-registered in OSF (https://osf.io/search/?q=tksh5&page=1)

## Background

The adverse experiences and profound structural changes related to the COVID-19 pandemic have resulted in severe psychosocial stress all over the globe.

There is a consensus that families have been particularly strained by the policies implemented to contain the virus, e.g., due to limited access to family support services [1] and daycare facilities/ nurseries or schools [2], which resulted in additional childcare responsibilities for parents while often having to work from home. While restriction measures have been gradually reduced or completely lifted, the long course of the crisis and the accumulation of stress factors such as existential fears and worries, loss and grief, economic disadvantages, negative personal and social changes in many life areas represent persistent stressors that can have adverse effects on e.g., mental health [3].

Accordingly, studies have found an increase in depression and anxiety symptoms among parents since the beginning of the pandemic [4, 5], with caregivers of young children at particular risk for these mental health issues [6]. There is also evidence for an increase of parenting stress [7–9], which is closely related to the quality of parent–child-interaction [10] and both parental and child mental health [10–12].

Child mental health soon became a matter of concern in the early stages of COVID-19. National and international studies found significant increases of psychological problems in children and adolescents [e.g. 13, 14] compared to pre-pandemic data. However, these studies largely focused on school-aged children or merely included younger children without highlighting their specific situation. Hence, comparable investigations of infants’ and young children’s mental health as well as their caregivers’ wellbeing are sparse- possibly due to this group being less directly affected by the structural restrictions (e.g., school closures) implemented to contain the virus. However, parental psychosocial stress during the different waves of the pandemic is likely to have impacted very young children: They depend almost exclusively on the physical and emotional care and protection of their parents in a phase of life characterized by rapid brain growth and development [15, 16], rendering them particularly vulnerable to stressful environmental influences [17] as evident during the pandemic—with severe possible implications for a healthy development [18, 19].

One of the very few investigations with a specific focus on infants and toddlers is the CoronabaBY study which surveys psychosocial stress factors such as parenting stress and parental and child mental health problems in families with 0–3 year olds in Germany [20]. First cross-sectional results from a high incidence phase in 2021 showed that infants and toddlers showed an overall similar level of mental health problems at this point compared to pre-pandemic studies. However, a substantial number of the surveyed parents struggled with affective symptoms and reported limited emotional resources for childcare due to high levels of parenting stress. Because young children’s mental health is generally closely linked to the psychosocial wellbeing of their caregivers [10, 11, 21–23], there could still have been delayed detrimental effects of parental stressors in later stages of the pandemic. Given that children’s mental health is the foundation for healthy development [24], it is of particular interest to understand the extent to which potentially harmful stressors were present in this population at different stages of the pandemic in order to provide appropriate support services following the very recent crisis.

We therefore investigated psychosocial stress factors, namely perceived pandemic burden, parenting stress, and parent and child mental health outcomes in German families with children aged 0–3 years in a repetitive cross-sectional study during three different phases of the COVID-19 pandemic in 2021/22 (waves I and III = phases of high incidence rates and restriction measures and wave II = low incidence rates and relaxations). We aimed to answer the following research questions:

1. How many families experienced psychosocial stress factors during phases of higher and lower COVID-19 incidence rates/ restriction measures during the CoronabaBY study?

We expected a higher prevalence of perceived pandemic burden, parenting stress, parental anxiety and depression symptoms, infant crying, sleeping, and feeding problems, and toddlers’ emotional and behavioral problems among families in survey wave I, due to high incidence rates and lockdown measures compared with families surveyed in wave II during the summer with low incidence rates and restrictions, followed by a rise in the number of psychosocially stressed families during the wave III survey period with its renewed steep increase in infection rates (emerging Omicron variant).

2. Which sociodemographic (e.g., income, education) and pandemic-related (e.g., restricted social support, increased family conflicts) markers might contribute to the aforementioned psychosocial stress factors?

## Methods

### Study design

The CoronabaBY study investigates intermediate and long-term psychosocial stress during high and low incidence phases of the COVID-19 pandemic (‘Corona’) in families with infants and toddlers (‘baby’) in Bavaria (Southern Germany) (‘BY’). As data has been collected continuously since 1st of February 2021, within a cross-sectional analysis we aimed to compare the number of psychosocially stressed families between the three different survey waves during the pandemic over a period from February 2021 to March 2022. The study protocol was approved by the Ethics committee of the Technical University of Munich (vote no. 322/20 S) and pre-registered in OSF (https://osf.io/search/?q=tksh5&page=1).

### Participants

All participants were recruited and surveyed via smartphone app “Mein Kinder- und Jugendarzt” (“My pediatrician”) (www.monks-aerzte-im-netz.de) which is a well-established communication tool connecting parents with their pediatrician. In a two-step recruitment procedure, all pediatricians in Bavaria using “My pediatrician” as part of their practice management were invited to participate in the study (*N* = 300). After giving informed consent (*N* = 73, response rate = 24.3%), an invitation for study participation was sent out via app to all eligible patients of the participating pediatricians. All parents of children between 3 months and 3 years who used the app and who understood the German study invitation were eligible to take part. Study invitation and detailed information were presented via app. Subsequent informed consent was also given via app. 18,531 study invitations were sent out via push-message. 3449 parents were included after giving informed consent and a remaining total of 2940 parents completed the study questionnaires.

### Measures

All data were collected by standardized questionnaires via app. Participants were asked questions on general sociodemographic characteristics, perceived pandemic burden, parenting stress and parent and child mental health outcomes.

#### Pandemic related restrictions and perceived pandemic burden

Overall, ten questions were asked about specific restrictions and perceived burden related to the pandemic (e.g., ‘During the strictest pandemic measures, how restricted did you feel with regard to social contacts?’). The perceived “pandemic burden” for parents and children was derived from the 5-point-answer (from 1 = *not at all stressful* to 5 = *very stressful*) to the global question: ‘Taken together, what do you think: How stressful is/was the COVID-19 pandemic for you (please think of measures like social restrictions but also your personal experiences, related worries etc.)?’ and ‘Taken together, what do you think: How stressful is/ was the COVID-19 pandemic for your child?’, respectively.

#### Parenting stress

To assess parenting stress, we applied the parent domain of the German Version of the ‘Parenting Stress Index (PSI)’ (“Eltern-Belastungs-Inventar” EBI; [25]). High scores indicated limited parental resources for upbringing and care for the child. The parent domain includes the following subscales: ‘health’ (parental health impairment as a cause or a result of parenting stress), ‘isolation’ (lacking integration in social networks), ‘role restriction’ (perceived limitations as a result of being a parent), ‘parental competence’ (parental doubt about their own abilities to manage upbringing and care for their child), ‘attachment’ (emotional relation of parent to the child), ‘depression’ (limited emotional availability within the parent-child-relationship) and ‘spouse related stress’ (as a result of being a parent). Answers were given on a 5-point Likert scale ranging from 1 = *strongly agree* to 5 = *strongly disagree* resulting in a possible score range of 28 to 140. The three cut-off categories for each subscale and the whole parent domain were ‘*not stressed’* (T-value < 60), ‘*stressed’* (T-value = 60–69), and ‘*strongly stressed’* (T-value ≥ 70). Internal consistency of the parent domain has been proven to be good (α = 0.93), and retest reliability after one year has been shown to be r = 0.87. Correlations with stress indicators and related constructs have resulted in the assumption of test validity [25, 26].

#### Parental depression and anxiety symptoms

Current parental depression and anxiety symptoms were assessed with the State-Trait-Anxiety-Depression Inventory (STADI; [27]). The questionnaire including four subscales (‘emotionality’, ‘worry’, ‘anhedonia’ and ‘dysthymia’) was answered on a 4-point scale ranging from 1 = *not at all* to 4 = *very much*, resulting in a possible score range of 20 to 80. Based on age- and sex-dependent standardized cut-off T-values, each domain (‘depression’, ‘anxiety’, ‘total’) was defined by symptoms to be ‘*far below average’* (T- value < 30)*, ‘below average’* (T-value = 30–39)*, ‘average’* (T-value = 40–60)*,* ‘*above average’* (T-value = 61–70), or ‘*far above average’* (T-value > 70). Internal consistency of the global State-Scales (α = 0.92), the State-Depression-Scale (α = 0.87) and the State-Anxiety-Scale (α = 0.90) have been proven to be good. Validity can be assumed based on comparison with other test procedures [28].

#### Infants’ crying, sleeping and feeding problems and toddlers’ emotional and behavioral problems

For infants (0–16 months), the two subscales ‘crying/whining/sleeping’ and ‘feeding’ of the Questionnaire for Crying, Sleeping and Feeding (CSF; [29]) were applied. Parents answered 38 questions on behaviors in their infants. Answers were given on 4-point-scales and mean values were calculated (ranging from 1 to 4). According to validated cut-off values, the dichotomous outcome *noticeable problems* and *no problems* were calculated for the domains ‘crying/whining/sleeping’ (cut-off value: 1.84, sensitivity: 87%, specificity: 92%) and ‘feeding’ (cut-off value: 1.27, sensitivity: 57%, specificity: 77%). The CSF also comprises questions to identify excessive crying as defined by the Wessel criterion (‘rule of threes’) [30]. The validity of the questionnaire has been secured by the proof of high internal consistencies of the scales as well as by correlations with behavior diaries and clinical diagnoses [29].

For toddlers (from 17 months old), the Strengths and Difficulties Questionnaire (SDQ, short form of the German Version; [31]) was used to examine emotional and behavioral problems. Parents were asked to classify the individual characteristics to be *not true*, *somewhat true* or *certainly true* for their child in four domains (‘emotional symptoms’, ‘conduct problems’, ‘hyperactivity/inattention’, and ‘peer relationship problems’), resulting in a score range of 0–40 points. Cut-off values indicated child behavior to be ‘*no problems’* (0–13 points), ‘*borderline’* (14–16 points) or *‘noticeable problems’* (17–40 points). Internal consistency has been shown to range between α = 0.73 and α = 0.86. By means of comparison with other corresponding scales (e.g., Child Behavior Checklist), the validity of the instrument can be assumed [32, 33].

### Statistical analyses

The present cross-sectional analysis is based on three survey waves of data collection: wave I: 1st of February to 7th of June 2021, wave II: 8th of June to 16th of October 2021, and wave III: 17th of October 2021 to 14th of March 2022. Selection and comparison of time periods was based on COVID-19 incidences and corresponding measures to contain the pandemic: wave I was characterized by high incidences and strict measures (e.g., closing of schools and close contact services until March, limited availability of first vaccination and a first occurrence of a COVID mutation). Wave II comprised the summer months with relatively low incidences, the opportunity to get vaccinated for everyone and relaxations of measures. Wave III was again characterized by very high incidences (emerging Omicron variant) but had fewer restrictions for vaccinated or recovered people compared to earlier high incidence phases in Bavaria, Germany.

Statistical differences between the sociodemographic characteristics of the three survey wave samples were detected by using Chi Squared test for categorical and ANOVA for continuous variables.

To answer the first research question, we calculated Chi Squared Tests and corresponding effect sizes (Phi coefficient φ) to detect potential differences of the addressed psychosocial stress factors between the samples of the three individual survey waves. To adjust for children’s age and sex, logistic regression models were calculated with survey wave and children’s age and sex as independent variable and the respective psychosocial outcome as dependent variable.

In order to obtain clinically relevant answers, the outcome variables were dichotomized as follows: Pandemic related restrictions/ changes and perceived pandemic burden were dichotomized into high/ very high (point 4 and 5 on 5-point Likert-scale) versus low perceived restrictions (points 1–3), respectively into stressful/ very stressful (point 4 and 5 on 5-point Likert-scale) compared to less stressful (points 1–3). Parenting stress (EBI) was classified into stressed/strongly stressed versus not stressed. Parental mental health problems (STADI) were dichotomized into above average/ far above average versus average/ below average/ far below average, and toddler’s emotional and behavioral problems (SDQ) into borderline/ noticeable problems versus no problems.

In a second step, we addressed the question, which factors might have contributed to the surveyed psychosocial stress factors, and included both sociodemographic factors and pandemic-related factors as potential predictors. To explore if these factors predicted parenting stress (EBI total score), maternal depression and anxiety symptoms (STADI total score, T-values), infants’ crying/whining/sleeping problems (total score of crying/whining/sleeping subscale), and toddlers’ emotional and behavioral problems (SDQ total score), four multiple linear regression models over all three survey waves were calculated: block-wise multiple linear regression models with binary sociodemographic variables as predictors in the first block (parental education status, parental financial status before the pandemic, financial burden due to pandemic, having siblings, child age, chronical illness/ disability of the child) and pandemic related variables in the second block (survey wave, restricted family support services, increased family conflicts, restricted parental social contacts and perceived pandemic burden) were conducted. The formation of the models resulted in the calculation of beta weights and their p-values for corresponding predictor variables. Requirements for calculating the multiple linear regression models were met.

For the linear regression models, independent variables were dichotomized as follows: Education status was dichotomized into *high* (university degree and high school diploma) and *low* (secondary and lower secondary school diploma). Financial status was also dichotomized into *high* (“large expenses possible” and “bigger additional expenses possible”) and *low* (“smaller additional expenses possible”, “little scope for additional expenses”, “additional expenses not possible”). Accordingly, financial burden due to the pandemic was dichotomized (yes: small, medium or huge financial burden versus no financial burden due to the pandemic). Chronic illness or disability of the child was defined as any chronic illness (also allergy, hyperactivity) and/or disability. Since submission of questionnaires was only possible when all items were completed, we had only a few missing values because of obvious misreporting of parental age.

All described results were based on an alpha level of 5%. A post-hoc Bonferroni correction was applied to control for multiple testing. Analyses were performed in IBM SPSS Statistics Version 28.0 for Windows.

## Results

### Sample characteristics

In total, we examined 2940 parent–child dyads, 1,004 of whom participated in wave I, 938 in wave II, and 998 in wave III (Table 1). Overall, 92.9% (n = 2731) of the surveyed parents were mothers with a mean age of 33.5 years (SD: 4.8), 6.6% fathers (mean age: 35.7 years, SD: 7.0), and 0.5% were “grandparents and others”. Children were on average 16.4 months old (SD: 11.7, range: 0–43 months) and were divided into ‘infants’ (n = 1404) with a mean age of 5.5 months (SD: 3.6) and ‘toddlers’ (n = 1536) with a mean age of 26.3 months (SD: 6.6).

**Table 1:** Sample characteristics

	Wave I (Feb. 21–June 21)	Wave II (June 21–Oct. 21)	Wave III (Oct. 21–March 22)
Parents % (n)			
Mothers	93.7 (941)	93.0 (872)	92.0 (918)
Born in Germany	91.5 (919)	90.7 (851)	91.6 (914)
Mother tongue German	92.8 (932)	91.2 (855)	92.1 (919)
Level of education			
University degree	41.6 (418)	41.6 (390)	44.8 (447)
High school diploma	18.4 (185)	18.2 (171)	18.5 (185)
Secondary school diploma	30.7 (308)	29.6 (278)	27.9 (278)
Lower secondary school diploma	8.3 (83)	9.8 (92)	8.1 (81)
Other qualifications	1.0 (10)	0.6 (6)	0.5 (5)
Financial status (before pandemic)			
Very large additional purchases possible	11.5 (115)	8.1 (76)	9.7 (97)
Large additional purchases possible	46.3 (465)	40.8 (383)	43.8 (437)
Small additional purchases possible	28.8 (289)	35.9 (337)	32.5 (324)
Very small additional purchases possible	5.9 (59)	5.8 (54)	5.7 (57)
No additional purchases possible	1.1 (119)	1.3 (12)	1.4 (14)
Not specified	6.5 (65)	8.1 (76)	6.9 (69)
Children			
Infants n	557	425	420
Infants M_age_	5.9 months * SD = 3.0	5.6 months SD = 3.5	4.9 months *, SD = 4.1
Toddlers n	439	507	571
Toddlers M_age_	25.8 months *, SD = 6.5	26.1 months, SD = 6.4	26.9 months *, SD = 6.7
Boys % (n)	51.0 (512)	53.6 (503)	53.2 (531)
Chronic illness and/or disability % (n)	8.1 (81)	9.0 (83)	7.3 (72)

*p ≤ 0.05 significant difference between survey wave I and survey wave III

### Perceived pandemic burden and pandemic-related restrictions

Almost two thirds of the parents perceived the pandemic as stressful or very stressful in wave I (Table 2). This proportion significantly decreased from wave I to wave II and remained on a high level in wave III. The frequencies of perceived individual pandemic-related restrictions can be seen in Table 2.

**Table 2:** Proportions of perceived high/ very high pandemic-related restrictions and burden calculated by Chi Squared Tests

Kind of restriction/change			Wave I (Feb. 21-June 21)	Wave II (June 21-Oct. 21)		Wave III (Oct. 21-March 22)	Wave I versus Wave II	Wave II versus Wave III	Wave I versus Wave III
			% (n)				Statistical significance ^b^ (Effect size φ)		
Participants perceiving a high/very high level of restrictions^a^ related to…									
Parent social contacts			75.1 (754)	32.6 (306)		41.1 (410)	* (−0.426)	* (0.088)	* (−0.345)
Child social contacts			63.6 (638)	27.4 (275)		30.7 (307)	* (−0.362)	n.s. (0.037)	* (−0.328)
Family support services			74.8 (751)	55.7 (523)		58.6 (585)	* (−0.200)	n.s. (0.029)	* (−0.172)
Leisure activities			93.3 (937)	59.3 (557)		68.1 (680)	* (−0.403)	* (0.091)	* (−0.320)
Participants perceiving a high/very high level of pandemic-related changes^a^									
Changes in childcare situation			42.3 (425)	28.6 (268)		25.1 (251)	* (−0.144)	n.s. (−0.039)	* (−0.182)
Increased family conflicts			16.5 (165)	15.6 (147)		16.1 (161)	n.s. ( 0.010)	n.s. (0.006)	n.s. (−0.004)
Worries about COVID- Infections			47.7 (479)	34.4 (320)		55.7 (555)	* (−0.138)	* (0.216)	* (−0.079)

Overall perceived pandemic burden	% (n)			Statistical significance (Effect size φ)		
Participants perceiving the pandemic as stressful/ very stressful^a^						
Parent	65.3 (656)	57.5 (539)	62.2 (621)	* (−0.082)	n.s. (0.049)	n.s. (−0.032)
Child (parent report)	36.3 (365)	34.5 (323)	35.0 (350)	n.s. (−0.02)	n.s. (0.007)	n.s. (0.013)

*p ≤ 0.05

^a^Based on responding options 4 and 5 on a 5-point-Likert scale, ^b^adjusted for children’s age and sex. Effect size φ: < 0.01 indicates a small effect, 0.3 a medium effect and 0.5 a large effect

### Parenting stress and parental mental health

Parenting stress was present in 38.2% of the parents in wave I. This proportion was slightly higher in wave II (46.1%, p = 0.09) and significantly higher in wave III (51.2%) compared to wave I (Table 3).

**Table 3:** Parenting stress (EBI parent domain), Parental and Child Mental Health Problems [STADI (parents), CSF (infants), SDQ (toddlers)]

	Wave I (Feb. 21–June 21)	Wave II (June 21–Oct. 21)	Wave III (Oct. 21–March 22)	Wave I versus Wave II	Wave II versus Wave III	Wave I versus Wave III
% (n)				Statistical significance^a^ (Effect size φ)		
Parenting stress (EBI)				Comparison of dichotomized data: not stressed versus stressed/ strongly stressed		
Not stressed	61.8 (609)	53.9 (493)	48.8 (474)	* (0.08)	n.s. (0.052)	* (0.131)
Stressed	30.2 (297)	33.4 (305)	39.8 (387)			
Strongly stressed	8.0 (79)	12.7 (116)	11.4 (111)			
Parental mental health problems (STADI)				Comparison of dichotomized data: far below/ below average/ average versus above/ far above average		
Mothers						
Far below average	3.9 (36)	3.6 (31)	3.7 (33)	n.s. (0.02)	n.s. (0.025)	n.s. (0.045)
Below average	11.4 (105)	12.5 (107)	11.4 (103)			
Average	60.4 (556)	57.9 (497)	56.7 (510)			
Above average	20.5 (189)	22.8 (196)	24.6 (221)			
Far above average	3.7 (34)	3.1 (27)	3.7 (33)			
Fathers						
Far below average	8.5 (5)	1.8 (1)	1.4 (1)	n.s. (−0.139)	n.s. (0.168)	n.s. (0.033)
Below average	8.5 (5)	14.3 (8)	10.8 (8)			
Average	57.6 (34)	69.6 (39)	59.5 (44)			
Above average	18.6 (11)	8.9 (5)	23.0 (17)			
Far above average	6.8 (4)	5.4 (3)	5.4 (4)			
Child mental health problems (CFS/ SDQ)						
Infant crying, feeding and sleeping problems (CSF)						
Excessive crying (rule of three)	1.8 (18)	2.5 (23)	2.4 (24)	n.s. (0.023)	n.s. (−0.002)	n.s. (0.021)
Crying/ Whining/ Sleeping	26.5 (148)	31.5 (134)	35.5 (149)	n.s. (055)	n.s. (0.042)	* (0.097)
Feeding	35.1 (196)	38.1 (162)	36.9 (155)	n.s. (0.031)	n.s. (−0.013)	n.s. (0.019)
Toddlers’ emotional and behavioral problems (Categorial evaluation of SDQ total score)				Comparison of dichotomized data: no problems versus borderline/ noticeable problems		
No problems	81.6 (363)	77.6 (398)	76.1 (440)			
Borderline	10.1 (45)	11.5 (59)	11.6 (67)	n.s. (0.049)	n.s. (0.017)	n.s. (0.066)
Noticeable problems	8.3 (37)	10.9 (56)	12.3 (71)			

*p ≤ 0.05

^a^Adjusted for children’s age and sex. Effect size φ: < 0.01 indicates a small effect, 0.3 a medium effect and 0.5 a large effect

EBI- German Version of Parenting Stress Index (‘Eltern-Belastungs-Inventar’), STADI: State-Trait-Anxiety-Depression Inventory, CFS: Questionnaire for Crying, Sleeping and Feeding, SDQ: Strengths and Difficulties Questionnaire

Maternal anxiety and depression symptoms were similar in all three waves and ranged between 24.2% in wave I and 28.3% in wave III (Table 3). In fathers, these symptoms ranged between 14.3% and 28.4% (Table 3).

### Child mental health (crying, sleeping and feeding, emotional and behavioral problems)

In wave I, 26.5% of the infants showed problems on the crying/whining/sleeping subscale of the CSF. In wave III, this proportion was significantly higher (35.5%, p = 0.009) (Table 3).

**Fig. 1:**
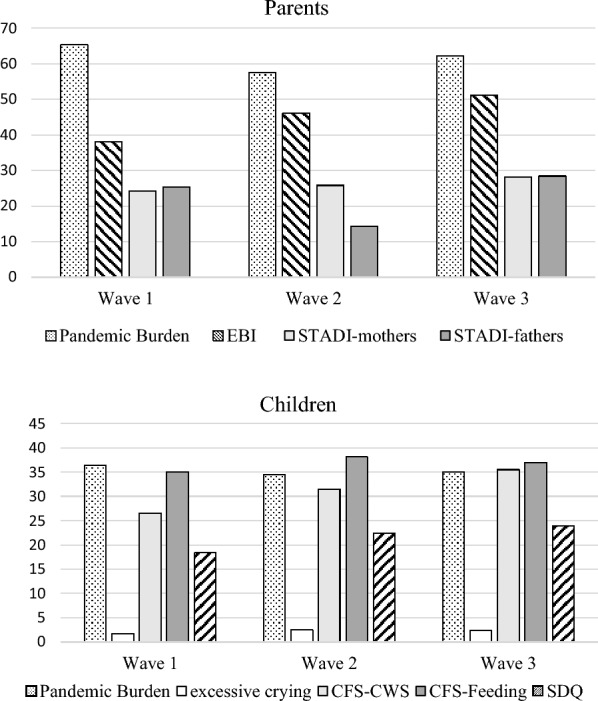
Percentage of Families with Psychosocial Stress Factors in each Survey Wave

In wave I, 18.4% of the toddlers showed at least borderline emotional and behavioral problems, in wave III the proportion was 23.9. Figure 1 shows the percentage of families with psychosocial stress factors in each survey wave (Fig. 1).

#### Influencing factors on parenting stress, maternal symptoms of depression and anxiety, infants’ crying/sleeping problems and toddlers’ emotional and behavior problems

The block-wise, fully adjusted linear regression model (*R*^*2*^ = 0.264, *F*(12, 2551) = 76.23, *p* < 0.001) showed familial conflicts to have the highest effect size (β = 0.355, p < 0.001) on the outcome parenting stress (EBI total score) (Table 4). For maternal symptoms of depression and anxiety (STADI total score) (*R*^*2*^ = 0.272, *F*(12, 2370) = 73.76, *p* < 0.001), increased familial conflicts had the highest effect size (β = 0.305, p < 0.001).

**Table 4:** Sociodemographic and pandemic-related predictors for EBI, STADI (mothers), Crying/Whining/Sleeping (CWS) score and SDQ^a^

Predictors	EBI total score				STADI total score (mothers)				CWS sub score				SDQ total score			
	B	SE B	ß	R^2^	B	SE B	ß	R^2^	B	SE B	ß	R^2^	B	SE B	ß	R^2^
First block				*0.053*				*0.083*				*0.032*				*0.093*
Child’s age (months)	0.210	0.034	0.122*		0.192	0.02	0.193**		0.000	0.003	0.002		0.059	0.020	0.074*	
More than one child^b^	1.583	0.798	0.039*		1.363	0.48	0.059*		−0.096	0.022	−0.126**		0.773	0.277	0.074*	
Child’s chronic illness and/or disability^b^	5.568	1.405	0.077**		2.412	0.802	.060*		0.062	0.046	0.038		3.087	0.425	0.189**	
Level of education^b^	5.280	0.820	0.127**		1.399	0.481	0.058*		0.069	0.022	−0.088*		−0.683	0.280	−0.065*	
Financial status (before pandemic)^b^	−5.38	0.805	−0.132**		−4.025	0.472	−0.171**		−0.086	0.022	−0.113**		−1.688	0.276	−0.162**	
Second block				0.264				0.272				0.095				*0.175*
Child’s age (months)	0.114	0.031	0.066**		0.150	0.018	0.151**		0.000	0.003	−0.002		0.05	0.02	0.064*	
More than one child^b^	−0.809	0.719	−0.020		0.053	0.430	0.002		−0.120	0.022	−0.159**		0.418	0.268	0.04	
Child’s chronic illness and/or disability ^b^	3.345	1.247	0.046*		0.981	0.724	0.024		0.059	0.045	0.036		2.612	0.411	0.16**	
Level of education^b^	4.711	0.733	0.113**		1.140	0.437	0.047*		0.059	0.022	0.075*		−0.742	0.272	−0.07*	
Financial status (before pandemic)^b^	−2.077	0.739	−0.051*		−2.093	0.441	−0.089**		−0.054	0.022	−0.071*		−1.007	0.276	−0.097**	
Survey Wave	2.821	0.444	0.117**		0.310	0.266	0.022		0.054	0.014	0.119**		0.300	0.165	0.048	
Financial burden due to pandemic	0.854	0.839	0.018		1.236	0.503	0.046*		−0.003	0.025	−0.003		0.911	0.308	0.078*	
Perceived pandemic burden	3.152	0.431	0.142**		2.333	0.259	0.181**		0.042	0.012	0.102**		0.421	0.164	0.073*	
Worries about COVID-Infections	1.568	0.297	0.096**		0.883	0.177	0.094**		0.000	0.009	0.001		0.295	0.113	0.07*	
Increased family conflicts	6.206	0.330	0.355**		3.065	0.196	0.305**		0.050	0.010	0.149**		0.958	0.121	0.216**	
Restrictions: family support services	1.558	0.348	0.088**		0.542	0.210	0.053*		0.029	0.011	0.084*		0.121	0.126	0.028	
Restrictions: parental social contacts	−1.530	0.394	−0.079**		−0.146	0.236	−0.013		0.000	0.012	−0.001		−0.192	0.145	−0.04	

N = 2581. Significance indicated by *p ≤ 0.05, **p ≤ 0.001

^a^Block-wise selection approach. ^b^Dichotomised

EBI- German Version of Parenting Stress Index (‘Eltern-Belastungs-Inventar’), STADI: State-Trait-Anxiety-Depression Inventory, CFS: Questionnaire for Crying, Sleeping and Feeding, SDQ: Strengths and Difficulties Questionnaire

For infants’ crying/sleeping problems (crying/whining/sleeping subscore of the CSF) the model (R^2^ = 0.095, F(12, 1237) = 10.79, p < 0.001) yielded having siblings to be a protective predictor with an effect size of β = − 0.159 (p < 0.001) whereas increased familial conflicts (β = 0.149, p < 0.001) was the risk factor with the highest effect size.

For toddlers’ emotional and behavioral problems (SDQ total score) the model (R^2^ = 0.175, F(12, 1356) = 23.97, p < 0.001) yielded increased familial conflicts (β = 0.216, p < 0.001) to have the highest effect size.

## Discussion

In a comparison of the three survey waves of the CoronabaBY study, including a total of 2940 parents with their children aged 0–3 years, we cross-sectionally investigated the number of families experiencing psychosocial stress factors in relation to higher and lower COVID-19 incidences and restrictions in Bavaria, Southern Germany. Our results show that psychosocial stress factors in families with infants and young children remained highly prevalent over the course of the pandemic. While parental high pandemic burden mirrored incidence rates and respective restriction measures, the number of caregivers experiencing noticeable parenting stress increased irrespective of pandemic events. Parental mental health problems were highly evident, but remained more or less stable during the course of the pandemic. Similarly, toddlers’ mental health problems were highly evident in later stages of the pandemic but did not increase significantly during the course of the study. In contrast, the number of infants with mental health problems significantly increased from one high incidence phase to another. Of several sociodemographic and pandemic-related variables, we found the increase of familial conflicts during the pandemic to have the strongest negative influence on parenting stress, maternal, infants’ and toddlers’ mental health.

Looking at our results in detail, we found a perceived high pandemic burden in up to 65% of our sample, which appears to be a slightly higher rate than in a German comparison study (59% with high perceived pandemic burden) including parents of children younger than 14 years [34]. This result corroborates findings that parents of young children are particularly vulnerable to experiencing the pandemic as stressful (e.g., [2]). Partly as expected, the number of families with a high pandemic burden roughly mirrored the restrictions during the individual survey waves with a prevalence peak during survey wave I, a significant decrease in survey wave II but only a slight renewed increase in survey wave III. These findings are in line with another study showing a peak of disease-related distress early during the virus outbreak and a decrease as time proceeded [35]*.*

A different picture emerged for parenting stress across the three survey waves: From the beginning of the study, with 38.2% of the families being affected, parenting stress was highly pronounced compared to pre-pandemic data (see [20]). Contrary to our initial assumption, the number of families with elevated parenting stress significantly increased in wave II despite lower COVID-19 incidences and fewer restrictions and only non-significantly rose to a peak in wave III. Although the effect sizes were small, overall more than half of the surveyed participants (51.2%) were affected by parenting stress.

While there are no studies that are fully comparable with regard to design, survey periods, and target group, a longitudinal study on parenting stress found an increase with a longer duration of the pandemic in the year 2020 [36]. In addition, a German longitudinal investigation of the general population found that psychological distress did not decrease between two high incidence phases in 2020 despite lesser restrictions during the second survey timepoint [37]. As parenting stress did not decrease despite relaxation of measures to contain the virus, it is likely to be a longer-term stress factor. Since it is closely related to parental [38–40] and child mental health problems [10–12] and negatively impacts the parent–child-relationship [38, 41], intervention efforts should aim at reducing parenting stress in families with young children in the aftermath of the pandemic.

Turning to parental mental health, we identified a peak of 28.3% of mothers and 28.4% of fathers with symptoms of depression and anxiety in survey wave III. The number of affected parents appeared to be higher compared to a pre-pandemic German study with approximately 20% of parents with children under the age of three experiencing affective symptoms [42]. Contrary to our assumptions, the number of parents with affective symptoms remained high almost irrespective of pandemic events, i.e., relaxation of measures. This result is roughly in line with an Austrian study on the general population showing a substantial persistence in mental health problems even 6 months after lifting of pandemic-related restrictions [43].

Taking a look at children’s mental health, the number of toddlers with borderline or noticeable emotional and/or behavioral problems rose from 18.4% in wave I to a maximum of 23.9% in wave III, however these increases were not significant. Still, while the number of toddlers with borderline and noticeable mental health problems almost exactly corresponded to the norm in survey wave I, this appears to be no longer the case in later periods of the pandemic [32, 44]. Prevalence rates for infants’ crying/sleeping, and feeding problems were already in the upper range compared to pre-pandemic data in survey wave I (compare [20]). The number of infants with excessive crying or feeding problems did not change significantly in relation to different survey periods. However, the proportion of infants with crying/sleeping problems was significantly higher in survey wave III than in survey wave I. One of the vanishingly small number of studies targeting infant crying, sleeping and feeding problems during the pandemic also used the CSF and found combined crying/sleeping but not feeding problems to be significantly higher prevalent in infants born during the pandemic [45]. While the small effect size in our study as well as differences in definition and operationalization of these problems have to be considered, with as much as 35.5% of infants affected, this rate is now higher than the ones found in pre-pandemic comparison studies [46]. These findings suggest a staggered negative impact of pandemic-related factors on young children’s mental health, and policy makers should be made aware that the needs of infants and toddlers are as relevant as those of e.g., school-aged children in the context of pandemic after-care.

In a second step, we explored possible influencing factors on parenting stress and mothers’ and child mental health problems. For all four outcomes, increased family conflicts had the highest predictive value, with a medium to large effect on parenting stress, a medium effect on maternal affective symptoms, a small to medium effect on toddlers’ emotional and behavioral problems and a small effect on infant crying/sleeping problems. This result highlights that also in the pandemic with its multiple external stressors, the family microclimate is particularly relevant for parental and child wellbeing and should be one focus when counseling burdened families, e.g., by providing conflict management strategies.

Individually, we found higher parental education to be predictive of higher parenting stress, which is in line with earlier findings of the CoronabaBY study and might be associated with higher educated parents worrying more about the pandemic (compare [20]). Survey wave and parental perceived pandemic burden also influenced parenting stress, however all effect sizes were small. Mothers’ symptoms of depression and anxiety were more pronounced with increasing child age. In contrast, a meta-analysis on moderating factors for maternal depression and anxiety during COVID-19 did not find child age to be influential [47]. As the found effect size in our sample of mothers was small, this result might not be of high practical relevance. Higher pandemic burden was also predictive for maternal mental health, again with a small effect size.

Infant crying/sleeping problems were predicted by survey wave and parental pandemic burden, whereas having a sibling was a protective factor. The latter result could be explained by parents’ greater experience in handling crying/sleeping problems when having more than one child and has also been shown by another study on infant crying/ sleeping problems during the pandemic in Germany [45]. Again, corresponding effect sizes in our study were small. Toddlers’ emotional and behavioral problems were slightly more pronounced for children with a chronic disease or a disability, which is line with earlier findings [48, 49].

There are several possible underlying causes for the trajectories of the surveyed psychosocial stress factors: First, the decreasing number of parents perceiving a high pandemic burden over time might reflect the reduced visibility of COVID-19 with the gradual relaxation of measures, but could additionally indicate the development of a habituation effect as a coping strategy. Second, given the permanence of COVID at the time of our survey, the increase in parenting stress over the course of the pandemic as well as the constantly high number of parents with mental health issues might be explained by lack of prospects, resulting in mental exhaustion—the so called ‘pandemic fatigue’ [37]. Third, with a longer duration of the pandemic—and again irrespective of pandemic events—the number of young children with deteriorated mental health has increased to a worrying level, while at the beginning of the study the numbers for both infants and toddlers still corresponded to the norm. As both parenting stress [10–12] and parental mental health issues [21–23] are known to be related to child mental health problems, the results corroborate the possibility of a staggered negative influence of high parental burden in this regard. However, these considerations remain theoretical as they cannot be answered on the basis of the data available in this study and will need to be addressed by future studies.

This study has strengths and limitations that have to be considered when interpreting the found results. The CoronabaBY study was the first and largest German investigation on psychosocial well-being during the pandemic specifically targeting families with children aged 0–3 years. Besides including a large number of participants, parenting stress and parental and child mental health were assessed with validated standardized psychological questionnaires without notable missing values. In addition, the individual survey periods as well as the whole study duration of 13 months were considerably longer than of many other comparison studies, enabling a broader perspective that goes beyond a momentary snapshot. Also, we investigated later stages of the pandemic while a majority of the pandemic-related research was conducted around the first outbreak of the virus.

We recruited a high number of financially well-off families with good education, German background, and mostly mothers (compare [20]). These aspects have to be considered with regard to the generalizability of our study results. Another limitation might be that the CoronabaBY study started a year after the initial outbreak of the virus and thus may not capture the most severe pandemic-related stress experiences. However, a significant proportion of the surveyed sample exhibited psychosocial stress factors, even if the crisis was no longer a new phenomenon, and the number of burdened parents and infants even increased during the course of the study. As the number of fathers in our sample was relatively small, we cannot provide specific results for them. Future studies should aim to shed light on fathers' mental health during and in the aftermath of the pandemic. Children’s mental health was assessed via parental report and we cannot rule out the probability that parents with higher stress levels perceive their child’s behavior as more troublesome. Finally, due to the cross-sectional nature of our data, we cannot draw causal conclusions with regard to the impact of the pandemic on the surveyed psychosocial stress factors. However, this study sheds light on trajectories of psychosocial stress in families with infants and toddlers in different stages of a global crisis and adds knowledge essential to provide adequate support for this vulnerable group.

## Conclusion

Taken together, our results show that psychosocial stress factors in families with infants and toddlers remained highly pronounced or even increased with a longer course of the pandemic - despite relaxation of restrictions and lower incidences. Although the pandemic might start to fade into the background in public perception, the ongoing support needs of families with young children must not be allowed to fall out of focus. Because of their particularly high vulnerability and susceptibility to parental burden, promoting infants' mental health has to be a top priority for policy makers and health professionals in the aftermath of COVID-19. Future investigations need to explore the underlying causes of the trajectories found and specifically examine the relationship between parental and child outcomes in a longitudinal design. Support measures should focus on promoting a positive microclimate in the family, e.g., through conflict counselling, by reducing parenting stress and by strengthening parental resources so that they can be fully available for the signals and needs of their young children.

## Data Availability

All data generated or analysed during this study are included in this published article.

## Abbreviations

CSF: Questionnaire for Crying, Sleeping and Feeding
EBI: Eltern-Belastungs-Inventar [German Version of ‘Parenting Stress Index (PSI)’]
SDQ: Strengths and Difficulties Questionnaire
STADI: State-Trait Anxiety-Depression Inventory
